# Cariprazine as monotherapy in a case of First Episode of Psychosis (FEP)

**DOI:** 10.1192/j.eurpsy.2024.1503

**Published:** 2024-08-27

**Authors:** P. Nasiou, D. Antoniadis

**Affiliations:** Psychiatric Hospital, Thessaloniki, Greece

## Abstract

**Introduction:**

First-Episode Psychosis (FEP) is a variable condition, characterized by the emergence of new psychotic features for a period of at least 1 week. ( Marques et al.. European Psychiatry 2016; 33 S258) The treatment is antipsychotic medications, which are generally divided into two categories: first and second generation antipsychotics, and they are dopamine antagonists or dopamine partial agonists.

**Objectives:**

The purpose of this presentation is to assess the efficacy of monotherapy treatment with cariprazine of the First Episode of Psychosis (FEP) in a young patient.

**Methods:**

A 19-year old man was involuntarily admitted to the psychiatric intensive care unit because of aggressive and inappropriate behaviour towards his mother including threats to kill her and exhibiting his genitals. His medical history included short periods of depressed mood, as well as physical symptoms such as loss of hair and gastrointestinal symptoms, since he was 18 years old.

When the patient was admitted he was cautious and anxious. During the interview he made reference to auditory hallucinations that commanded him to sexually stimulate himself in front of his mother and also persecutory delusions. Upon admission his total PANSS score was 127. The positive subscale score was 21. The patient was treated with monotherapy cariprazine, gradually increasing the dose from 1,5 mg to 6 mg per day . Furthermore, he was adjunctively treated with sertraline, gradually increasing the dose from 50 to 150 mg.

**Results:**

After a period 24 days since admission the patient clinically improved and was discharged. His total PANSS score was 73 and the positive subscale was 9. He suffered no adverse effects from his treatment.

**Conclusions:**

The use of cariprazine as a treatment for a FEP of a young male significantly improved his PANSS score after a 24-day treatment and also his disorganised behaviour. Of note, rapid tranquilization was avoided. According to the literature this is considered satisfactory response to treatment (Leucht et al. Schizophr Res. 2005; 79:231-8.). Nevertheless further investigation on the efficacy of the particular medication is necessary as its use is relatively recent in the treatment of psychosis. (Garnock et al. CNS Drugs. 2017; 31:513-525)

**Disclosure of Interest:**

None Declared

